# The Non-Dipping Blood Pressure Phenotype Is Independently Linked to Systemic Inflammation and Atherogenic Burden

**DOI:** 10.3390/jcm15062374

**Published:** 2026-03-20

**Authors:** Mert Deniz Savcilioglu, Kemal Ozan Lule, Yunus Cakalli, Mehmet Murat Sucu

**Affiliations:** 1 Cardiology Department, Faculty of Medicine, Gaziantep University, Gaziantep 27410, Türkiye; yunus.cakalli@gmail.com (Y.C.); mmuratsucu@gmail.com (M.M.S.); 2 Internal Medicine Department, Faculty of Medicine, Gaziantep University, Gaziantep 27410, Türkiye; drkemalozanlule@gmail.com

**Keywords:** non-dipping phenotype, ambulatory blood pressure monitoring, SIRI, atherogenic index of plasma, inflammation, circadian blood pressure pattern

## Abstract

**Background:** A non-dipping blood pressure pattern, defined as an insufficient nocturnal decline in systolic blood pressure, is associated with increased cardiovascular risk and target organ damage. While metabolic abnormalities contribute to circadian blood pressure dysregulation, the independent roles of systemic inflammation and atherogenic burden in individuals without overt metabolic disease remain insufficiently characterized. We aimed to evaluate the associations of systemic inflammatory response index (SIRI) and atherogenic index of plasma (AIP) with nocturnal blood pressure pattern. **Methods:** This retrospective cross-sectional study included 469 adults who underwent 24 h ambulatory blood pressure monitoring (ABPM) at a single tertiary cardiology outpatient clinic. Participants were classified as dippers (≥10% nocturnal systolic BP decline) or non-dippers (<10%). Hierarchical logistic regression models were constructed sequentially: Model 1 (age, sex), Model 2 (Model 1 + AIP, fasting glucose), and Model 3 (Model 2 + SIRI). Discriminative performance was assessed using receiver operating characteristic (ROC) analysis. **Results:** Non-dipping was present in 62.5% of participants. LDL cholesterol, AIP, and SIRI were higher in non-dippers, whereas CRP was higher in dippers. In hierarchical regression, AIP was independently associated with non-dipping in Model 2 (OR = 3.672, *p* = 0.003). After addition of SIRI, SIRI remained independently associated (OR = 1.913, *p* < 0.001), and model explanatory power increased (Nagelkerke R^2^ = 0.104). AIP, fasting glucose (inverse association), and age also remained significant. Individual discrimination was modest for SIRI (AUC = 0.572) and AIP (AUC = 0.576), while the multivariable model achieved an area under the curve (AUC) of 0.660. Non-dipping prevalence increased across SIRI quartiles (*p* for trend = 0.009). **Conclusions:** Both inflammatory and atherogenic burden were associated with a non-dipping blood pressure pattern in individuals without overt metabolic disease. Although the discriminative capacity was modest, combined inflammatory–metabolic assessment may provide additional biological insight into circadian blood pressure dysregulation.

## 1. Introduction

Hypertension is a leading determinant of cardiovascular mortality worldwide and affects approximately 1.3 billion adults globally. Importantly, a substantial proportion of hypertensive individuals do not present with overt diabetes or advanced cardiometabolic disease [1,2]. This underscores the importance of exploring biological mechanisms underlying blood pressure dysregulation independent of established metabolic comorbidities.

Ambulatory blood pressure monitoring (ABPM) offers prognostically significant insights beyond average blood pressure readings by delineating circadian blood pressure patterns [3,4]. Under physiological circumstances, systolic blood pressure decreases by around 10% during the night. The absence or attenuation of this decrease, characterized as a non-dipping pattern, correlates with increased cardiovascular events, target organ damage, and arterial stiffness [4,5,6].

Non-dipping, which signifies sustained nighttime vascular strain, has been associated with autonomic dysregulation, endothelial dysfunction, and increased sympathetic nervous system activity. Furthermore, chronic, low-level inflammation has become a key factor in the development of hypertension, potentially affecting the regulation of vascular tone and the circadian patterns of blood pressure [7,8,9,10]. Composite inflammatory indices, including systemic immune-inflammation index (SII) and systemic inflammatory response index (SIRI), have been linked to cardiovascular mortality and hypertensive phenotypes. Compared with other leukocyte-derived indices, SIRI incorporates monocytes, which play a central role in vascular inflammation and endothelial dysfunction. Increased inflammatory indices have been observed in individuals exhibiting non-dipper and reverse-dipper phenotypes [11,12,13,14]. Nevertheless, the independent impact of inflammatory burden, independent of metabolic factors, is still unclear.

Atherogenic index of plasma (AIP), which is the logarithm of the triglyceride to HDL cholesterol ratio, indicates a lipoprotein profile that promotes atherosclerosis [15].

AIP has been associated with cardiovascular risk and subclinical atherosclerosis, and prior studies have suggested a relationship between AIP and non-dipping [15,16]. Nevertheless, the combined and independent roles of inflammatory and atherogenic burden in circadian blood pressure disruption have not been sufficiently investigated.

Therefore, we evaluated the associations of SIRI and AIP with nocturnal blood pressure patterns in a cohort without overt diabetic or advanced metabolic comorbidity and examined the incremental contribution of inflammatory burden using hierarchical regression analysis.

## 2. Materials and Methods

### 2.1. Study Design and Population

This cross-sectional study was conducted at the Cardiology Outpatient Clinic of Gaziantep University Şahinbey Research and Practice Hospital. Adult patients who underwent 24 h ABPM as part of routine clinical evaluation were consecutively included.

### 2.2. Inclusion Criteria

Participants were eligible for inclusion if they were between 18 and 65 years of age and underwent ABPM for the evaluation of suspected hypertension in the absence of previously established cardiometabolic disease. Eligible individuals had no prior diagnosis of hypertension and no known chronic systemic diseases such as diabetes mellitus, hyperlipidemia, or rheumatologic disorders. In addition, participants were required to have ABPM findings compatible with elevated blood pressure or hypertension according to current guideline definitions [4], with complete ABPM reports including mean daytime and nighttime blood pressure values and circadian blood pressure patterns. To ensure temporal consistency between laboratory parameters and blood pressure measurements, complete laboratory evaluations—including complete blood count and biochemical parameters—obtained on the same day as ABPM were required for inclusion.

### 2.3. Exclusion Criteria

Patients were excluded if they had a prior diagnosis of hypertension; evidence of acute infection or active inflammatory disease at the time of evaluation; active malignancy or ongoing oncological treatment including chemotherapy, radiotherapy, or immunotherapy; pregnancy; chronic inflammatory or autoimmune diseases such as rheumatoid arthritis, systemic lupus erythematosus, or inflammatory bowel disease; a history of hematological disorders including hematologic malignancies, severe anemia, thrombocytopenia, or thrombocytosis; acute kidney injury or end-stage renal disease; use of corticosteroids, immunosuppressive agents, or cytotoxic medications during the study period; insufficient or unreliable ABPM recordings; missing key laboratory parameters required for the analysis (including complete blood count, lipid profile, or biochemical measurements); a history of major surgery, acute cardiovascular events, or significant trauma within the preceding three months; or previously diagnosed diabetes mellitus or dyslipidemia in order to minimize the confounding effects of established metabolic disease.

A total of 624 individuals were screened during the study period. Patients with incomplete clinical records, missing laboratory data, or inadequate ABPM recordings were excluded. After applying the exclusion criteria, 469 participants were included in the final analysis. The study was conducted in accordance with the principles of the Declaration of Helsinki and approved by the Gaziantep University Non-Interventional Clinical Research Ethics Committee (21 January 2026; decision no: 2026/65). Due to the retrospective nature of the study and the use of anonymized hospital records, institutional permission for the use of hospital data was obtained from the hospital administration.

### 2.4. Ambulatory Blood Pressure Monitoring

Twenty-four–hour ABPM was performed using a validated oscillometric device (Borsam Echo BP Holter^®^, Borsam Biomedical Instrument Co., Shenzhen, China). Blood pressure measurements were automatically obtained at 15 min intervals during daytime and at 30 min intervals during nighttime. Participants were instructed to maintain their usual daily activities but to keep the monitored arm still during measurements. Daytime and nighttime periods were defined according to participants’ self-reported wake and sleep times. Mean daytime and nighttime systolic and diastolic blood pressure (SBP and DBP) values were automatically calculated using the device software (eCardio V7.0). ABPM recordings with less than 80% valid measurements were excluded from the analysis. Nocturnal systolic blood pressure decline was calculated as the percentage reduction in mean nighttime systolic blood pressure relative to mean daytime systolic blood pressure as follows:

Nocturnal SBP decline (%) = [(Mean daytime SBP − Mean nighttime SBP)/Mean daytime SBP] × 100.

According to current guideline recommendations, patients were classified as dippers when nocturnal systolic blood pressure reduction was between 10% and 20%, and as non-dippers when the reduction was <10%. Individuals with extreme dipping (>20% decline) or reverse dipping (<0% decline) were excluded from the analysis to maintain a more homogeneous comparison between the two primary circadian blood pressure patterns [3,4].

### 2.5. Laboratory Measurements

Venous blood samples were obtained after overnight fasting at the time of outpatient evaluation. Hematological parameters were measured using an automated complete blood count analyzer(Sysmex XN-1000, Sysmex Corporation, Kobe, Japan), and biochemical parameters were analyzed in the hospital’s central laboratory using standard methods. To minimize the potential influence of acute inflammatory conditions, individuals with C-reactive protein (CRP) levels ≥ 10 mg/L were excluded from the analysis.

### 2.6. Inflammatory and Atherogenic Indices

Systemic inflammatory and atherogenic indices were calculated using hematological and lipid parameters derived from fasting venous blood samples obtained during outpatient evaluation. SIRI was calculated as the product of neutrophil and monocyte counts divided by the lymphocyte count. The SII was calculated as the platelet count multiplied by the neutrophil count and divided by the lymphocyte count. The aggregate index of systemic inflammation (AISI) was derived by multiplying neutrophil, monocyte, and platelet counts and dividing the resulting value by the lymphocyte count. All hematological parameters were expressed as absolute cell counts (×10^9^/L) and were obtained from automated complete blood count analysis. AIP was calculated as the base-10 logarithm of the triglyceride-to-HDL cholesterol ratio according to the following formula:

AIP = log_10_(Triglyceride/HDL cholesterol).

Triglyceride and HDL concentrations originally measured in mg/dL were converted to mmol/L before AIP calculation using standard conversion factors (triglycerides: mg/dL ÷ 88.57; HDL: mg/dL ÷ 38.67). Among these indices, SIRI and AIP were considered the primary variables of interest in the present study.

SIRI = (Neutrophil × Monocyte)/Lymphocyte

SII = (Platelet × Neutrophil)/Lymphocyte

AISI = (Neutrophil × Monocyte × Platelet)/Lymphocyte

### 2.7. Statistical Analysis

Statistical analyses were performed using IBM SPSS Statistics (Version 22, IBM Corp., Armonk, NY, USA). Continuous variables were assessed for normality using visual inspection of histograms and probability plots together with skewness and kurtosis values. Given the relatively large sample size, minor deviations from normality were considered acceptable. Normally distributed variables were expressed as mean ± standard deviation, whereas non-normally distributed variables were presented as median (interquartile range).

Comparisons between dipper and non-dipper groups were performed using the independent samples *t*-test for normally distributed variables and the Mann–Whitney U test for non-normally distributed variables. Categorical variables were compared using the chi-square test.

Pearson correlation analysis was applied to evaluate associations between night SBP decline (%) and normally distributed variables, whereas Spearman’s rank correlation analysis was used for non-normally distributed variables.

Hierarchical binary logistic regression analysis was performed to identify variables independently associated with a non-dipping blood pressure pattern. Non-dipping status (dipper = 0, non-dipper = 1) was entered as the dependent variable. Variables were entered sequentially according to conceptual domains and based on clinical relevance and univariable findings:

- Model 1 (Demographic model): age and sex;
- Model 2 (Metabolic model): Model 1 + AIP and fasting glucose;
- Model 3 (Inflammation model): Model 2 + SIRI.

Results were expressed as odds ratios (ORs) with 95% confidence intervals (CIs). The model fit was assessed using the Omnibus test for model coefficients, and its explanatory power was evaluated with Nagelkerke R^2^. To check for multicollinearity, variance inflation factors (VIFs) were calculated from a linear regression model that included all independent variables. No significant multicollinearity was found, as all VIF values were below 1.3.

An examination of the receiver operating characteristic (ROC) curve was performed using projected probabilities obtained from the logistic regression models to assess discriminative performance. The area under the curve (AUC) was calculated along with 95% confidence intervals.

Additionally, individuals were classified into quartiles according to their SIRI and AIP levels. The distribution of dipping status across these quartiles was further analyzed using the chi-square test, and linear trend analysis was used to assess possible dose–response correlations.

A two-tailed *p*-value < 0.05 was considered statistically significant.

## 3. Results

A total of 469 participants were included in the final analysis, of whom 176 (37.5%) were classified as dippers and 293 (62.5%) as non-dippers. Baseline characteristics of the study population are summarized in Table 1. Blood pressure status did not differ significantly between groups (χ^2^ = 0.901, *p* = 0.343). Age, fasting glucose, HbA1c, creatinine, neutrophil count, HDL cholesterol, and uric acid levels were comparable between dipper and non-dipper groups (all *p* > 0.05).

CRP levels were significantly higher in dippers compared with non-dippers (4.11 ± 3.05 vs. 3.51 ± 2.06 mg/L, *p* = 0.011). LDL cholesterol was significantly higher in non-dippers than in dippers (133.41 ± 32.23 vs. 127.23 ± 28.76 mg/dL, *p* = 0.037). Similarly, AIP values were significantly elevated in non-dippers (0.213 ± 0.225 vs. 0.157 ± 0.229, *p* = 0.009). Among inflammatory indices, SIRI was significantly higher in the non-dipper group (1.27 [1.09–2.10] vs. 1.23 [0.75–1.86], *p* = 0.009), whereas SII and AISI did not differ significantly between groups (*p* = 0.186 and *p* = 0.096, respectively). (Table 1).

Spearman correlation analysis was performed to evaluate the relationships between demographic, metabolic, inflammatory, and blood pressure parameters (Table 2). Correlation analysis revealed generally weak associations between night SBP decline (%) and the evaluated clinical and laboratory parameters. Significant negative correlations were observed between night SBP decline and neutrophil count (r = −0.096, *p* = 0.039), SII (r = −0.112, *p* = 0.015), and SIRI (r = −0.104, *p* = 0.025). No significant correlations were identified between night SBP decline and age (r = −0.029), fasting glucose (r = 0.020), HbA1c (r = −0.066), creatinine (r = 0.003), CRP (r = −0.040), LDL cholesterol (r = −0.078), HDL cholesterol (r = −0.022), uric acid (r = −0.028), AIP (r = −0.078), or AISI (r = −0.083) (all *p* > 0.05). Overall, the magnitude of the correlation coefficients indicated small effect sizes across all associations.

**Figure 1:**
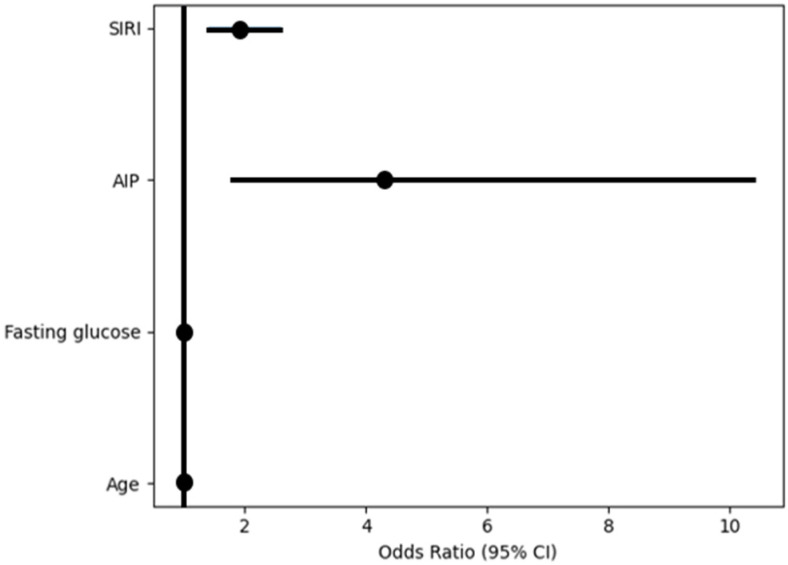
Forest plot showing variables independently associated with the non-dipping blood pressure pattern in the final hierarchical logistic regression model (Model 3). Odds ratios (ORs) with 95% confidence intervals for variables included in the final model are displayed. The vertical reference line indicates an OR of 1. Values to the right of the line represent a higher likelihood of the non-dipping phenotype. AIP: atherogenic index of plasma; SIRI: systemic inflammatory response index; CI: confidence interval.

Hierarchical logistic regression analysis was performed to evaluate factors independently associated with a non-dipping blood pressure pattern (Table 3). In Model 1, which included demographic variables, neither age nor sex was significantly associated with non-dipping status, and the overall model was not significant (Omnibus *p* = 0.290; Nagelkerke R^2^ = 0.007). After the addition of metabolic variables in Model 2, both AIP and fasting glucose were independently associated with non-dipping status. Higher AIP was associated with a higher likelihood of non-dipping (OR = 3.672, 95% CI: 1.546–8.721, *p* = 0.003), whereas higher glucose levels were associated with a lower likelihood of non-dipping (OR = 0.985, 95% CI: 0.972–0.998, *p* = 0.022). Age also became significantly associated with non-dipping status (OR = 1.018, 95% CI: 1.002–1.034, *p* = 0.025). The explanatory power increased to Nagelkerke R^2^ = 0.044 (Omnibus *p* = 0.002). In Model 3, which additionally included SIRI, SIRI was independently associated with non-dipping status (OR = 1.913, 95% CI: 1.411–2.595, *p* < 0.001). AIP (OR = 4.300, 95% CI: 1.776–10.408, *p* = 0.001), fasting glucose (OR = 0.982, 95% CI: 0.968–0.995, *p* = 0.007), and age (OR = 1.017, 95% CI: 1.001–1.034, *p* = 0.041) remained significantly associated with non-dipping status, whereas sex was not associated. The final model showed improved explanatory power (Nagelkerke R^2^ = 0.104; Omnibus *p* < 0.001). Model calibration was adequate across all models based on the Hosmer–Lemeshow goodness-of-fit test (all *p* > 0.05) (Table 3). The independent associations identified in the final regression model are illustrated in a forest plot (Figure 1).

ROC curve analysis was performed to evaluate the discriminative ability of inflammatory and metabolic indices for identifying non-dipping blood pressure pattern (Table 4). SIRI showed modest discriminative performance with an AUC of 0.572 (95% CI: 0.521–0.623, *p* = 0.009). Similarly, AIP demonstrated modest discriminative ability with an AUC of 0.576 (95% CI: 0.522–0.629, *p* = 0.006).

**Figure 2:**
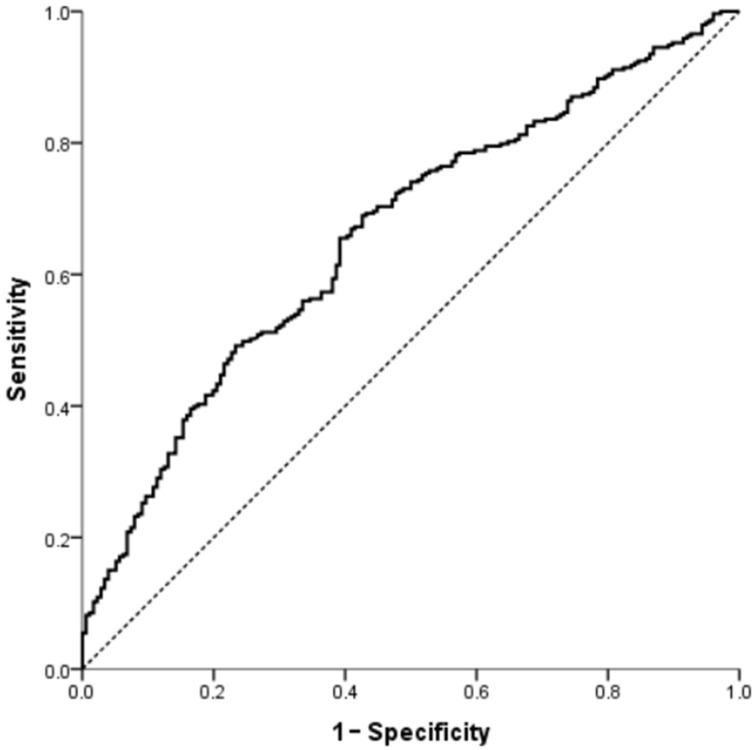
ROC curve of the multivariable logistic regression model for predicting non-dipping blood pressure pattern. The diagonal dashed line represents the line of no discrimination (AUC = 0.5).

The multivariable model including age, sex, AIP, fasting glucose, and SIRI showed improved discriminative performance with an AUC of 0.660 (95% CI: 0.611–0.710, *p* < 0.001) (Figure 2).

Overall, the multivariable model demonstrated higher discriminative ability compared with individual indices.

To assess the distribution of dipping status, participants were further categorized based on SIRI quartiles. The prevalence of non-dipping exhibited an upward trend across the SIRI quartiles; the highest proportion was found in the fourth quartile (81.4%), while the second quartile had the lowest (47.8%). Significant differences were observed across the overall group (Pearson χ^2^ = 32.55, *p* < 0.001). Furthermore, a significant linear trend was identified, suggesting an increased prevalence of non-dipping with elevated SIRI levels (*p* for trend = 0.009) (Table 5).

The prevalence of non-dipping blood pressure pattern across AIP quartiles is shown in Table 6. The proportion of non-dipper patients increased progressively from the lowest to the highest AIP quartile (55.2% in Q1, 58.0% in Q2, 67.5% in Q3, and 69.2% in Q4). Although the overall association between AIP quartiles and dipping status did not reach statistical significance (Pearson χ^2^ *p* = 0.066), a significant linear trend was observed across increasing AIP quartiles (*p* = 0.010)

## 4. Discussion

Our findings suggest that the non-dipping blood pressure pattern should not be viewed merely as an isolated disturbance of circadian rhythm. Inflammatory activation may contribute to nocturnal blood pressure dysregulation through several mechanisms, including endothelial dysfunction, oxidative stress, and increased sympathetic nervous system activity. These mechanisms may impair vascular relaxation during nighttime and attenuate the physiological nocturnal decline in blood pressure. Rather, it appears to reflect a more complex vascular phenotype characterized by increased AIP and SIRI.

### 4.1. Limited Contribution of the Demographic Model

In Model 1, age and sex did not significantly explain the non-dipper phenotype. While prior research has demonstrated a correlation between non-dipping prevalence and advancing age, and the 2023 ESH guidelines indicate a reduction in circadian blood pressure variability with age [3,4], age alone did not prove to be the primary factor within our study group. This finding indicates that non-dipping is not only a result of vascular aging, and it probably encompasses other biological processes as well.

### 4.2. Metabolic Model: The Function of AIP and the Inverse Relationship with Glucose

Within Model 2, AIP demonstrated an independent association with the non-dipping pattern. The current finding is consistent with previous research that has linked AIP to non-dipper hypertension [17,18].

AIP serves as an indicator of an atherogenic lipoprotein profile, specifically small dense LDL particles, which are linked to endothelial dysfunction [15,16,18]. Consequently, diminished nitric oxide bioavailability and heightened vascular tone could potentially impede the expected nocturnal decline in blood pressure.

The inverse association between fasting glucose and non-dipping warrants cautious interpretation. The modest protective signal observed in regression analysis does not imply a true beneficial effect. Rather, this finding may be related to the relatively narrow glycemic range within this metabolically uncomplicated cohort or to complex interactions between glucose metabolism and lipid-related vascular stress. In this context, AIP emerged as the more robust metabolic correlate of circadian disruption.

### 4.3. Inflammatory Model: SIRI’s Independent Role

The addition of SIRI to Model 3 enhanced the model’s overall explanatory capacity, and SIRI continued to demonstrate an independent association with non-dipping [13,14,17]. This supplementary effect reinforces the hypothesis that systemic inflammatory activation could play a role in circadian blood pressure dysregulation, independent of demographic and metabolic influences.

Cell-based inflammatory indices have previously been linked to cardiovascular mortality and resistant or reverse-dipper hypertension phenotypes [8,9,10,19]. Our findings extend this literature by demonstrating an independent association between SIRI and the categorical non-dipping phenotype in a population without overt metabolic disease.

Although correlation coefficients between nocturnal SBP decline and inflammatory markers were small, this does not represent a methodological inconsistency. Correlation analysis evaluates linear relationships with continuous variables, whereas the non-dipper classification represents a threshold-based clinical phenotype. It is plausible that inflammatory burden exerts a nonlinear or threshold-dependent influence on circadian blood pressure regulation. SIRI belongs to a broader group of hematology-derived inflammatory indices, including the neutrophil-to-lymphocyte ratio (NLR), platelet-to-lymphocyte ratio (PLR), and SII, which have been increasingly investigated as markers of systemic immune activation in cardiovascular diseases.

### 4.4. Interpretation of CRP Findings

CRP levels were unexpectedly higher in the dipper group. However, individuals with CRP ≥ 10 mg/L were excluded to minimize the influence of acute inflammatory states. CRP is an acute-phase reactant reflecting short-term hepatic inflammatory response, whereas SIRI integrates neutrophil, monocyte, and lymphocyte counts and may better represent chronic low-grade immune activation [10,19].

Given that non-dipping is associated with persistent nocturnal vascular stress rather than acute inflammatory events, the absence of a positive association with CRP may be biologically consistent. Moreover, the lack of correlation between CRP and nocturnal SBP decline suggests that CRP may be less sensitive to circadian hemodynamic alterations compared with composite leukocyte-based indices.

### 4.5. Balanced Evaluation of ROC Findings

Both SIRI and AIP demonstrated modest discriminative performance (Table 4). The increase in AUC in the multivariable model indicates that combined inflammatory and metabolic assessment provides more information than individual markers alone. Nevertheless, the overall discrimination remains limited, and these indices should not be interpreted as standalone diagnostic tools.

### 4.6. Isolated Hypertension Perspective

The absence of overt diabetic burden in our cohort allows interpretation within a phenotype closer to isolated hypertension. Global data indicate that a substantial proportion of hypertensive individuals do not have established cardiometabolic disease [1,2]. Our findings suggest that inflammatory and atherogenic processes may accompany circadian blood pressure disruption even before the development of advanced metabolic abnormalities.

### 4.7. Clinical Implications

The present findings suggest that the non-dipping blood pressure pattern may reflect more than a circadian variation detected by ambulatory monitoring and could represent an underlying vascular risk state characterized by inflammatory and atherogenic burden. In this context, indices such as SIRI and AIP, which can be easily derived from routine laboratory parameters, may provide insight into the biological background of the non-dipper phenotype. Although their individual predictive performance was modest, the combined evaluation of these indices may help characterize underlying vascular stress in patients with non-dipping hypertension, particularly in individuals without overt metabolic disease. Given their low cost and wide availability, SIRI and AIP may contribute to a more integrated approach to cardiovascular risk assessment. Recognition of the non-dipping phenotype may have important therapeutic implications in the management of hypertension. A blunted nocturnal decline in blood pressure has been consistently associated with increased cardiovascular risk, greater target organ damage, and worse long-term outcomes in hypertensive populations. Consequently, ambulatory blood pressure monitoring plays a central role in identifying abnormal circadian blood pressure patterns and improving cardiovascular risk stratification in clinical practice [6,20]. Beyond macrovascular complications, circadian blood pressure dysregulation has also been linked to microvascular injury. Previous investigations have demonstrated that hypertensive patients with a non-dipping pattern may exhibit retinal microvascular alterations, including reduced retinal capillary density independent of mean ambulatory blood pressure levels [21]. Moreover, abnormalities in circadian blood pressure patterns have been associated with structural retinal changes such as thinning of the retinal nerve fiber layer, suggesting early microvascular damage in patients with newly diagnosed hypertension [22]. These findings support the concept that abnormal nocturnal blood pressure patterns may reflect broader vascular and microvascular dysfunction rather than merely a variation in daily blood pressure rhythm. In this context, the associations observed in our study between the non-dipping phenotype and markers reflecting inflammatory and atherogenic burden may provide additional biological insight into the vascular mechanisms underlying circadian blood pressure dysregulation. Therefore, recognition of abnormal nocturnal blood pressure patterns may help guide more individualized antihypertensive management strategies and contribute to earlier identification of hypertension-related vascular injury.

### 4.8. Study Limitations

Several limitations should be acknowledged. First, the retrospective and cross-sectional design precludes causal inference and limits the ability to establish temporal relationships between inflammatory burden and the non-dipping blood pressure pattern. Second, the single-center nature of the study may restrict the generalizability of the findings.

Third, important cardiometabolic variables such as body mass index (BMI) and smoking status were not available and therefore could not be included in the multivariable adjustment. Residual confounding due to unmeasured clinical variables cannot be excluded. In particular, BMI and smoking status are well-recognized determinants of circadian blood pressure variation and may partly influence the observed associations.

Fourth, information on obstructive sleep apnea (OSA) and sleep quality—both known to significantly influence circadian blood pressure patterns—was not available in the dataset. Given that OSA is one of the strongest drivers of the non-dipper phenotype, the potential interaction between sleep-disordered breathing and inflammatory markers may represent an unmeasured source of confounding. Fifth, circadian blood pressure patterns were assessed using a single 24 h ABPM recording. Because dipping status may vary between recordings, repeated ABPM measurements might provide a more stable assessment of circadian blood pressure patterns.

Finally, the SIRI and AIP indices were derived from single baseline laboratory measurements, which may not fully reflect temporal variability in inflammatory or metabolic status. Future prospective multicenter studies with repeated biomarker and ABPM assessments are warranted to confirm these findings and clarify the causal relationships between inflammatory burden, atherogenic lipid profiles, and circadian blood pressure dysregulation.

## 5. Conclusions

In this study, a non-dipping blood pressure pattern was associated with increased atherogenic burden and systemic inflammatory activation. These findings suggest that the non-dipper phenotype may be linked to underlying vascular and inflammatory processes rather than representing only a circadian variation in blood pressure.

However, the discriminative ability of the evaluated indices was modest. Therefore, these markers should be interpreted cautiously and not as standalone diagnostic indicators. The combined assessment of inflammatory and atherogenic burden may provide additional insight into the biological background of nocturnal blood pressure dysregulation. Further prospective studies are required to better clarify the clinical implications of these associations.

## Figures and Tables

**Table 1:** Baseline Characteristics According to Dipping Status.

Variable	Dipper (*n* = 176)	Non-Dipper (*n* = 293)	*p*-Value
**Blood pressure status *n* (%)**			0.343
Elevated BP	93 (52.8)	168 (57.3)	
Hypertension	83 (47.2)	125 (42.7)	
Age (years)	49.14 ± 13.22	51.02 ± 12.94	0.130
Glucose (mg/dL)	98.81 ± 16.01	96.89 ± 16.04	0.209
HbA1c (%)	5.67 ± 0.55	5.65 ± 0.50	0.561
Creatinine (mg/dL)	0.76 ± 0.17	0.75 ± 0.17	0.508
CRP (mg/L)	4.11 ± 3.05	3.51 ± 2.06	**0.011**
Neutrophil (10^3^/µL)	5.59 ± 1.99	5.41 ± 2.00	0.343
LDL (mg/dL)	127.23 ± 28.76	133.41 ± 32.23	**0.037**
HDL (mg/dL)	48.75 ± 11.18	47.51 ± 10.93	0.238
Uric acid (mg/dL)	5.31 ± 1.41	5.50 ± 1.43	0.163
AIP	0.157 ± 0.229	0.213 ± 0.225	**0.009**
SII	624.9 (382.5–850.8)	583.0 (450.0–890.0)	0.186
AISI	380.3 (267.7–528.5)	337.9 (308.3–520.0)	0.096
SIRI	1.23 (0.75–1.86)	1.27 (1.09–2.10)	**0.009**

Continuous variables are presented as mean ± standard deviation or median (interquartile range), as appropriate. Categorical variables are expressed as number (percentage). Group comparisons were performed using the independent samples *t*-test for normally distributed variables, the Mann–Whitney U test for non-normally distributed variables, and the chi-square test for categorical variables. CRP values ≥ 10 mg/L were excluded to minimize the potential influence of acute inflammatory conditions. BP, blood pressure; CRP, C-reactive protein; LDL, low-density lipoprotein; HDL, high-density lipoprotein; AIP, atherogenic index of plasma; SII, systemic immune-inflammation index; AISI, aggregate index of systemic inflammation; SIRI, systemic inflammation response index. Statistically significant associations are shown in bold (*p* < 0.05).

**Table 2:** Correlations Between Night SBP Decline (%) and Clinical Parameters (*n* = 469).

Variable	r
Age	−0.029
Glucose	0.020
HbA1c	−0.066
Creatinine	0.003
CRP	−0.040
Neutrophil count	**−0.096**
LDL	−0.078
HDL	−0.022
Uric acid	−0.028
AIP	−0.078
SII	**−0.112**
AISI	−0.083
SIRI	**−0.104**

Pearson correlation coefficients are presented for normally distributed variables, and Spearman’s rho for non-normally distributed variables. Statistically significant associations are shown in bold (*p* < 0.05). Abbreviations: CRP: C-reactive protein; HbA1c: glycated hemoglobin; LDL: low-density lipoprotein; HDL: high-density lipoprotein; AIP: atherogenic index of plasma; SII: systemic immune-inflammation index; AISI: aggregate index of systemic inflammation; SIRI: systemic inflammatory response index.

**Table 3:** Hierarchical Logistic Regression Analysis for Non-Dipping Blood Pressure Pattern.

Variable	Model 1 OR (95% CI)	*p*	Model 2 OR (95% CI)	*p*	Model 3 OR (95% CI)	*p*
Age (years)	1.011 (0.997–1.026)	0.131	1.018 (1.002–1.034)	**0.025**	1.017 (1.001–1.034)	**0.041**
Sex (male)	0.919 (0.628–1.344)	0.663	0.859 (0.581–1.271)	0.448	0.798 (0.533–1.195)	0.274
AIP	—	—	3.672 (1.546–8.721)	**0.003**	4.300 (1.776–10.408)	**0.001**
Glucose (mg/dL)	—	—	0.985 (0.972–0.998)	**0.022**	0.982 (0.968–0.995)	**0.007**
SIRI	—	—	—	—	1.913 (1.411–2.595)	**<0.001**
Nagelkerke R^2^	0.007		0.044		0.104	
Omnibus *p*	0.290		0.002		<0.001	

Values are presented as odds ratios (ORs) with 95% confidence intervals (CIs). Model 1 included demographic variables (age and sex). Model 2 additionally included metabolic variables AIP and fasting glucose). Model 3 additionally included SIRI. Statistically significant associations are shown in bold (*p* < 0.05). Model performance was evaluated using Nagelkerke R^2^ and the Omnibus test of model coefficients. Abbreviations: OR, odds ratio; CI, confidence interval; AIP, atherogenic index of plasma; SIRI, systemic inflammation response index.

**Table 4:** ROC analysis for non-dipping blood pressure pattern.

Variable	AUC	95% CI	*p*-Value
SIRI	0.572	0.521–0.623	**0.009**
AIP	0.576	0.522–0.629	**0.006**
Multivariable model	0.660	0.611–0.710	**<0.001**

Values are presented as area under the curve (AUC) with 95% confidence intervals (CI). Statistically significant *p*-values are shown in bold. The multivariable model included age, sex, atherogenic index of plasma (AIP), fasting glucose, and systemic inflammation response index (SIRI). Abbreviations: AUC, area under the curve; CI, confidence interval; ROC, receiver operating characteristic; AIP, atherogenic index of plasma; SIRI, systemic inflammation response index.

**Table 5:** Distribution of dipping status according to SIRI quartiles.

SIRI Quartile	Dipper *n* (%)	Non-Dipper *n* (%)	Total
Q1	40 (33.9)	78 (66.1)	118
Q2	60 (52.2)	55 (47.8)	115
Q3	54 (45.8)	64 (54.2)	118
Q4	22 (18.6)	96 (81.4)	118

Pearson χ^2^ = 32.55, *p* < 0.001, *p* for trend = 0.009. Abbreviations: SIRI: systemic inflammation response index.

**Table 6:** Association Between AIP Quartiles and Non-Dipping Blood Pressure Pattern.

AIP Quartile	Dipper *n* (%)	Non-Dipper *n* (%)	Total
Q1	52 (44.8)	64 (55.2)	116
Q2	50 (42.0)	69 (58.0)	119
Q3	37 (32.5)	77 (67.5)	114
Q4	37 (30.8)	83 (69.2)	120

Pearson χ^2^ *p* = 0.066, Linear trend *p* = 0.010. Abbreviations: AIP: Atherogenic Index of Plasma.

## Data Availability

The datasets generated and/or analyzed during the current study are not publicly available due to institutional data protection policies but are available from the corresponding author on reasonable request.

## Abbreviations

The following abbreviations are used in this manuscript:

ABPM	Ambulatory Blood Pressure Monitoring
AIP	Atherogenic Index of Plasma
AISI	Aggregate Index of Systemic Inflammation
AUC	Area Under the Curve
BMI	Body Mass Index
BP	Blood Pressure
CBC	Complete Blood Count
CI	Confidence Interval
CRP	C-Reactive Protein
ESH	European Society of Hypertension
HDL	High-Density Lipoprotein Cholesterol
LDL	Low-Density Lipoprotein Cholesterol
OR	Odds Ratio
OSA	Obstructive Sleep Apnea
ROC	Receiver Operating Characteristic
SBP	Systolic Blood Pressure
SII	Systemic Immune-Inflammation Index
SIRI	Systemic Inflammatory Response Index
VIF	Variance Inflation Factor
